# School and class closures and adolescent mental health during the second and later waves of the COVID-19 pandemic in Finland: a repeated cross-sectional study

**DOI:** 10.1186/s12889-023-17342-8

**Published:** 2023-12-06

**Authors:** Arja Rimpelä, Heidi Kesanto-Jokipolvi, Anna Myöhänen, Lauri Heikonen, Sanna Oinas, Raisa Ahtiainen

**Affiliations:** 1 Faculty of Social Sciences, Department of Health Sciences, Tampere University, Tampere, Finland; 2 Faculty of Educational Sciences, Department of Education, University of Helsinki, Helsinki, Finland; 3 Faculty of Educational Sciences, Department of Teacher Education, University of Turku, Turku, Finland; 4 Department of Adolescent Psychiatry, Tampere University Hospital, Tampere, Finland

**Keywords:** Adolescents, Anxiety, COVID-19, Duration of school closure, Health complaints, Mental health, School

## Abstract

**Background:**

Since the start of the COVID-19 pandemic, several studies have shown deterioration of adolescents’ mental health when comparing periods before and after the start of the pandemic when there were national school closures. Less is known of the following waves with more variation in school closures and their duration. We study here, if variation in school or class closures was related to adolescents’ mental health, if the duration mattered, and if the relationships were gender sensitive.

**Methods:**

All comprehensive schools in Finland were invited to participate. Students (grades 7–9, age 13–16 years) answered digitally in November–December 2020 (n = 41,041) and April–May 2021 (n = 28,501). The responses were given anonymously. Mental health was measured by daily health complaints and moderate/severe anxiety (GAD-7, only in 2021). School and class closures were combined to a variable (yes/no). The duration of a closure was analysed in weeks. Logistic regression analysis was used.

**Results:**

In 2020, 14% of pupils reported a school closure and 33% in 2021. The gender-adjusted odds for daily health complaints were higher among those with the school or class closure compared to those without (OR = 1.2 (1.1–1.3) in 2020; OR = 1.3 (1.2–1.3) in 2021). For anxiety, the corresponding OR was 1.3 (1.2–1.4). Girls had higher odds for both measures than boys and the non-binary gender had the highest. A one-week increase in the duration of closure had a small effect on daily health complaints OR = 1.05 (1.02–1.09) in 2020; OR = 1.05 (1.02–1.08) in 2021) and anxiety OR = 1.05 (1.01–1.08). Gender differences in the associations of the school or class closure with mental health were negligible.

**Conclusions:**

A specific negative influence of school or class closures on adolescents’ mental health was seen when comparing those who had school/class closure and those who did not during the further waves of the pandemic. The duration of closure had a small effect, too. The non-binary gender had lowest mental health, but the influence of school closure on mental health was mainly similar between the genders. School closures are one of the factors in adolescents’ mental health, but not the only one.

## Introduction

When the WHO declared COVID-19 as a pandemic on 11 March 2020 countries all over the world closed schools and restricted physical contacts [1]. In Finland, schools were closed for two months in March-May 2020 and reopened for two weeks before the summer vacation in the end of May. During the summer, the epidemic situation stayed calm but worsened in October and stayed higher during the spring 2021 [2]. In international comparisons, the epidemic burden was, however, low in Finland. No nationwide school closure was implemented any more during the second and later waves of the pandemic, but based on local epidemic situation, temporary local and regional school closures of varying durations continued under the guidance of local governments and the Regional State Administrative Agencies [1]. Depending on a school, the duration of school closures for individual pupils varied. Some schools organized normal classroom teaching while others were forced to close their premises for fixed periods of time or to apply remote learning to part of the pupil groups (certain classes, grades) to avoid physical contacts and probability of transmissions. Consequently, individual pupils were exposed to different duration of school closures due to their place of residence. Using our nationwide surveys for upper grade (7–9) of comprehensive school pupils in November–December 2020 and April–May 2021, we investigate whether the variation in the duration of school/class closure is related to the variation in pupils’ mental health.

Since the beginning of the pandemic, several reviews have reported on mental health effects on children and youth. Most reviews cover studies from the early phase of the pandemic in 2020, some reach to the beginning of 2021 [3–9], and an umbrella review until the end of 2021 [10]. As summarised in the umbrella review, most reviews and individual studies show a consistent picture of lowering mental health wellbeing during the pandemic [10]. High prevalence of anxiety, depression, sleep disorders, suicidal behaviour, stress-related disorders, attention-deficit/hyperactivity disorder as well as other problems of mental health were high during the pandemic compared to findings in studies before the pandemic. Even though the results were parallel, the authors pointed out that most studies were cross-sectional and used non-representative samples. Little is known if the duration of school closure is related to mental health problems. Among general population, a systematic review and meta-analyses showed that days of lockdown did not moderate the association between the exposure to the pandemic and mental health symptoms [11].

It is well known that girls report more symptoms than boys, e.g., [12]. This was also true in most above-mentioned studies dealing with gender, but some studies showed no gender difference [13]. A longitudinal study suggests that the effects on the pandemic may differ between genders [14]; depressive and internalising symptoms increased during the pandemic among female but not among male adolescents. No study reported mental health results for a non-binary gender, but it has been reported that non-binary pupils experienced remote learning conditions overall more negatively compared to girls and boys [15].

We study here (a) if adolescents whose school or class had been closed reported more health complaints and anxiety compared to those whose school or class did not have that experience, and (b) if health complaints and anxiety increased with the increasing duration of school or class closure. Further, we are interested in differences between genders (boys, girls, non-binary). Our study population covers grade-levels 7 to 9 of comprehensive schools in Finland, surveyed in November–December 2020 and April–May 2021.

## Methods

### Participants

This study started in April 2020 when local and national education authorities were contacted for school surveys related to learning and wellbeing effects of the COVID-19 pandemic. A collaboration agreement was made with 16 municipalities first, after which the Ministry of Education and Culture supported the extension to the entire country. Research permits were obtained from the 16 municipalities and from the Ministry for the rest of the country. School leaders (principals) were contacted for school-level research permits. The survey for all comprehensive schools was repeated twice, In November–December 2020 and in April–May 2021. The present study uses data from these two surveys. The surveys were carried out during the same academic year. Partially, the same individuals participated in both surveys. The samples were not independent and were analysed separately.

The data were collected online using a digital survey system (Qualtrics). The links to the electronic survey were delivered to pupils by school principals who used the electronic communication channels of the schools. Participation was voluntary, which was explicitly mentioned in the instruction letter. Parents were informed by a digital letter, and they could prevent the participation of their child. Most pupils answered the survey during their school hours, but it was also possible to fill the questionnaire at home. The data were collected anonymously, but pupils were informed that school and municipality identification codes were included in the response.

In this study, responses from the upper grade-levels 7–9 (age 13–16 years) were used. In 2020, 41,041 seventh to ninth graders from 420 schools responded representing 60% of the 692 comprehensive schools with respective grade-levels. The schools represented 191 (62%) of the 310 Finnish municipalities. In 2021, there were 28,501 respondents from 321 schools representing 46% of the 687 schools representing 161 (52%) municipalities. Thus, the non-response occurred largely at school level due to school principals not distributing the survey. The sample distributions are presented in Tables 1 and 2.

### Outcome variables

#### Daily health complaints

Respondents were asked if they had had the following health complaints during the present term: (1) neck, shoulder, or back pain, (2) headache, (3) difficulties in falling asleep or getting awake night-time, (4) tiredness or exhaustion, (5) feeling low or depression, and (6) difficulties with concentration. These and corresponding questions have been widely used in measuring adolescent health and wellbeing. The symptoms are perceived, and they are not supposed to be diagnostic criteria. The underlying assumption is that at this age adolescents can assess and report their symptoms and feelings reliably. The items 1 to 3 and the item 5 have been used in the HBSC study [16] the item 4 e.g., in the nationwide School Health Promotion Study in Finland [17] and the item 6 in the Strength and Difficulties Scale [18]. For each symptom the options were: ‘seldom or not at all’, ‘approximately once a month’, ‘approximately weekly’, or ‘daily’. The time frame ‘during the present term‘ was used instead of usual ‘six months‘ to cover the whole school term and not the time during the breaks. In Finland, school term lasts about 4.5 months in autumn and 5 months in spring. If a participant had given an answer to at least one of the six items, possible missing answers in the other symptoms were replaced with the value ‘seldom or not at all’. If none of the items was answered, the respondent was excluded from the analyses. Cronbach’s alpha of the six health complaint questions was 0.82 in 2020 and 0.85 in 2021, which is on a very good level. If the respondent had answered ‘daily’ in at least one of the symptom questions, they were classified as ‘yes’ in the binary outcome variable *daily health complaints*, otherwise ‘no’. The distributions at both time points are presented in Table 1.

#### Moderate or severe anxiety

Anxiety during the last two weeks was measured only in 2021 with a generalized anxiety disorder scale (GAD-7). Its validity in a Finnish adolescent population has been good [19]. The items were (1) feeling nervous, anxious, or on edge, (2) not being able to stop or control worrying, (3) worrying too much about different things, (4) trouble with relaxing, (5) being so restless that it is hard to sit still, (6) becoming easily annoyed or irritable, (7) feeling afraid as if something awful might happen. The answer options were ‘not at all’ (0 points), ‘on several days’ (1 point), ‘on most of the days’ (2 points), ‘nearly every day’ (3 points), which were summarised to a scale of 0–21. Participants, who had answered five or more of the seven items, were included. If a respondent had given only five or six answers, the missing items were replaced by the respondent’s mean of the other items. Cronbach’s alpha of the anxiousness items was 0.92.

According to the instructions of GAD-7 measure the respondents were classified as ‘no’ (< 10), ‘moderate’ (10–14), or ‘severe’ (15–21), by their score. The distribution of this classification is presented in Table 1. For the analyses of this study, the classification was reduced binary. Therefore, the ‘no’ group remained, and the ‘moderate’ and ‘severe’ groups were combined into the ‘yes’ group of the binary outcome variable *moderate or severe anxiety*.

### Explanatory variables

#### School or class closure

Pupils were asked, if their class or school had been closed during the present term with the following items (more than one could be answered): (1) no, (2) no, but some other class from my school has been, (3) yes, my class or group has been in quarantine, (4) yes, my school has been in quarantine or closed, (5) yes, my class or group is now in quarantine. We first composed a binary variable *school or class closure* with options ‘no’ or ‘yes’. If respondents had ticked the item 1 or 2, they were classified as ‘no’. After that, respondents who had ticked the items 3, 4, or 5 were classified as ‘yes’. Distributions are presented in Table 1. To analyse the reliability of pupils’ answers, we calculated the percentage of students giving the same answer (yes or no) for each school in 2020 data. Because a school could have more than one building and all pupil groups did not necessarily answer at the same time, we did not expect a 100% concordance. When we accepted a 10% deviation of the yes/no answers in the school, 85% of the schools were placed into the range, which can be interpreted as a good concordance.

#### Duration of school or class closure

In the items 3 and 4, a further open question was presented for those who had ticked the previous item: how many days. For the item 5, the open question was: how many days it is expected to last. The durations were calculated separately from the open questions in the items 3, 4, and 5. In 2020, the digital data collection tool accepted only numbers. The analysis revealed a high number of missing answers, likely because closures, particularly if long, were difficult to express and remember in days. The text answers in 2021 were manually converted to days, considering the varying units the participants had given, e.g., weeks, months. The variables *duration of school/class closure* for each item in both years were classified and expressed in weeks. Each item was analysed separately because they were partly overlapping, and different types of closures were of interest. The distributions are presented in Table 2.

To exclude incorrect answers, we computed the maximum duration of closure which was the difference between the respondent’s answer date and the starting date of the school term. The personal maximums varied between 89 and 124 days in 2020, and between 95 and 130 days in 2021. Answers exceeding the maximum and answers reporting zero were excluded. In 2020, the number of excluded participants was N = 35 in item 3, N = 63 in item 4, and N = 26 in item 5. For 2021, the corresponding numbers were N = 63 in item 3, N = 76 in item 4, and N = 38 in item 5.

*Gender* was queried with three options: girl, boy, other. The last option is labelled as non-binary in the text and tables.

### Statistical methods

To investigate the possible group effect of the school, we computed intraclass correlations (ICC) for both outcome variables, categorised as described above, using school as a grouping variable. For the daily health complaints, the results were ICC = 0.0090 in 2020 and ICC = 0.014 in 2021 and for moderate or severe anxiety ICC = 0.0037. Due to the low ICC values, modelling of the school effect was omitted in the analyses [20]. In the computing procedure of ICC values, linear mixed effects models were used for each outcome variable by lme4 library of R [21], using school as a grouping variable without other predictors. In these models, ICC values were computed as a quotient of group level variance and the sum of group level and individual variances.

Logistic regression analysis was used for modelling the associations between the explanatory and outcome variables. Daily health complaints and moderate or severe anxiety were used as dependent variables. Gender and school or class closure or duration of school closure were added at the same time into the model as independent variables. To investigate the moderating effect between gender, we calculated interaction terms between gender and school closure / duration of school closure for each model.

All the analyses were computed by R Statistics [22], utilizing haven [23], psych [24], lme4 [21] and lubridate [25] libraries, except the interaction models mentioned in the text by IBM SPSS version 29.0.1.0(171).

## Results

Distributions of the variables are presented in Tables 1 and 2.

**Table 1:** Descriptive statistics in 2020 and 2021

Variable	2020		2021	
	n	%	n	%
**Gender**				
Boy	17,442	42.5	12,610	44.3
Girl	22,384	54.6	14,864	52.3
Non-binary	1201	2.9	963	3.4
Total	41,027	100.0	28,437	100.0
Missing	14		64	
**Daily health complaints**				
No	25,613	64.3	16,045	62.4
1–6	14,204	35.7	9656	37.6
Total	39,817	100.0	25,701	100.0
Missing	1224		2800	
**Anxiety** ^**a**^				
No (< 10)			20,408	80.4
Moderate (10–14)			2906	11.4
Severe (15–21)			2066	8.1
Total			25,380	100.0
Missing			3121	
**School or class closure**				
No	35,042	86.1	18,645	66.6
Yes	5669	13.9	9352	33.4
Total	40,711	100.0	27,997	100.0
Missing	330		504	

^a^Not measured in 2020

**Table 2:** Duration (weeks)^a^, mean (M) and SD^b^ of school or class closure in 2020 and 2021

Closure	2020			2021		
	n	Duration (weeks) M (SD)	Missing	n	Duration (weeks) M (SD)	Missing
Own group or class	1671	1.7 (1.9)	1036	2869	3.0 (2.2)	1120
School	1416	3.5 (3.5)	1979	4687	4.2 (2.2)	1544
Present	175	2.5 (2.9)	178	329	3.3 (2.6)	558

^a^Question presented only for those who reported a closure

^b^SD = standard deviation

Table 3 shows the odds ratios for daily health complaints and moderate or severe anxiety. In both years, the odds for daily health complaints were statistically significantly higher among those whose school or class had been closed compared to those with no closure. Correspondingly, the ORs for moderate or severe anxiety were statistically significantly higher for those with school or class closure.

Girls reported more symptoms and anxiety than boys, and those who perceived themselves non-binary, had especially high odds. The difference between boys and girls was larger in anxiety than in health symptoms and a particularly high OR was observed for those who identified themselves neither a boy nor a girl. The interaction terms of gender and school/class closure in the models for daily health complaints were not statistically significant (p = 0.765 in 2020 and p = 0.924 in 2021) showing that the effect did not differ between the genders. In the model for anxiety, the interaction term was significant (p = 0.024). However, in the gender-specific analyses, ORs varied only a little between the genders: Girls OR 1.2 (1.1–1.3), p < 0.001, N = 13,435; Boys OR 1.5 (1.3–1.7), p < 0.001, N = 10,798; Non-binary OR 1.3 (0.99–1.7), p = 0.056, N = 831.

**Table 3:** Odds ratios (95% CI) for health complaints and anxiety by school or class closure and gender

Variables in the model	Daily health complaints^a^				Moderate or severe anxiety^a,b^	
	2020 n = 39,493		2021 n = 25,365		2021 n = 25,064	
	OR (95% C.I.)	*p*	OR (95% C.I.)	*p*	OR (95% C.I.)	*p*
**School or class closure**						
No	1.0		1.0		1.0	
Yes	1.2 (1.1–1.3)	0.000	1.3 (1.2–1.3)	0.000	1.3 (1.2–1.4)	0.000
**Gender**						
Boy	1.0		1.0		1.0	
Girl	2.5 (2.4–2.6)	0.000	2.9 (2.7- 3.0)	0.000	3.6 (3.3–3.9)	0.000
Non-binary	6.0 (5.3–6.9)	0.000	6.1 (5.3–7.1)	0.000	7.9 (6.8–9.1)	0.000

^a^Model: school or class closure + gender

^b^Not measured in 2020

The associations of daily health complaints with the duration of school or class closure, expressed in weeks, are presented in Table 4 for both years. The ORs measure an increase in symptoms when the duration increases by one week. In 2020, the ORs for daily health complaints were low but reached the statistical significance in the models of ‘own group or class’, and ‘school’. The effects were, however, very small. For those who had present school or class closure, the OR was not significant. In 2021, only ‘school’ was statistically significant, and the OR was just marginally increased, but the OR was very low. The gender differences followed the same pattern as in the previous Table 3. The interaction terms of gender and duration of school or class closure were not statistically significant; in 2020 for models of ‘own class or group closed’, ‘school closed’ and ‘present closure’ the p-values were 0.565, 0.764, and 0.381 and in 2021, p = 0.602, p = 0.393, p = 0.669, respectively.

Table 5. presents the results for severe or moderate anxiety with the duration of school or class closure measures. Only the measure ‘school’ was statistically significant. The gender pattern was similar as in the earlier tables. The interaction terms were not statistically significant between the duration of closure measures and gender in these models (for ‘own group or class’ p = 0.899, for ‘school’ p = 0.704, for ‘present closure’ p = 0.339.)

**Table 4:** Odds ratios^a^ (95% CI) for daily health complaints by duration of closure in 2020 and 2021

2020												
**Variables in the model**	Own class or group closed^b^ n = 1581				School closed^b^ n = 1308				Present closure^b^ n = 141			
	OR	95% C.I.		*p*	OR	95% C.I.		*p*	OR	95% C.I.		*p*
**Duration of closure (weeks)**	1.07	1.01	1.13	0.018	1.05	1.02	1.09	0.004	1.13	0.98	1.30	0.095
Gender												
Boy	1.0				1.0				1.0			
Girl	2.3	1.8	2.8	0.000	3.0	2.3	3.8	0.000	0.7	0.3	1.5	0.339
Nonbinary	4.3	2.4	7.8	0.000	8.0	4.1	16.6	0.000	3.6	0.8	19.1	0.095
**2021**												
Variables in the model	Own class or group closed^b^ n = 2480				School closed^b^ n = 4144				Present closure^b^ n = 253			
	OR	95% C.I.		*p*	OR	95% C.I.		*p*	OR	95% C.I.		*p*
**Duration of closure (weeks**)	1.01	0.98	1.05	0.479	1.05	1.02	1.08	0.001	1.09	0.98	1.22	0.115
Gender												
Boy	1.0				1.0				1.0			
Girl	3.2	2.7	3.8	0.000	3.0	2.6	3.4	0.000	2.5	1.4	4.4	0.001
Non-binary	5.9	3.7	9.4	0.000	7.7	5.4	11.1	0.000	3.2	0.9	12.0	0.071

^a^OR expresses the increase in health complaints when the duration increases by one week. Question on duration presented only for those who reported a closure

^b^Model: Duration of closure + gender

**Table 5:** Odds ratios^a^ (95% CI) for moderate and severe anxiety by duration of closure in 2021

Variables in the model^a^	Own class or group closed^b^ n = 2448				School closed^b^ n = 4102				Present closure^b^ n = 252			
	OR	95% C.I.		*p*	OR	95% C.I.		*p*	OR	95% C.I.		*p*
**Duration of closure (weeks)**	1.00	0.95	1.05	0.954	1.05	1.01	1.08	0.008	1.05	0.92	1.18	0.473
**Gender**												
Boy	1.0				1.0				1.0			
Girl	3.7	3.0	4.7	0.000	3.4	2.9	4.1	0.000	2.8	1.4	5.5	0.003
Non-binary	8.2	5.1	13.0	0.000	8.1	5.7	11.4	0.000	2.4	0.5	9.7	0.224

^a^OR expresses the increase in anxiety when the duration increases with one week. Question on duration presented only for those who reported a closure

^b^Model: Duration of closure + gender

## Discussion

Our study showed higher daily health complaints and severe or moderate anxiety among those adolescents who were exposed to school or class closures during the second and later waves of the COVID-19 pandemic. A slight decrease in mental health was seen when the duration of closure increased. The non-binary gender had lower mental health compared to girls, and particularly to boys, but the influence of the school or class closure and duration of closure on mental health did not differ negligible between the genders. At the time of our study, there were no national school lockdown anymore but local and regional school or class closures of a varying duration.

Most previous studies have shown worsening of adolescent mental health during the pandemic compared to the time before (e.g., [12]). We showed that during the pandemic, mental health was worse among pupils whose school or class was closed compared to those without this experience. Results of this study indicate that school may represent security and stability under uncertain situations, such as the pandemic. Moreover, school provides a place to interact with peer, which is shown to be especially important during pandemic [17].

It is difficult to separate the influence of school closures from the influence of other pandemic restrictions and from the threat of the pandemic in people’s mind in general, but it is likely that our results reflect a specific effect of school closure more reliably than those comparing wellbeing before and after the start of the pandemic. In Sweden, schools were not closed for children under 16 years unlike in Finland [26] but nevertheless, a decrease in adolescents’ mental health was observed [27]. It seems that among adolescents both school closures and other aspects of the pandemic have influenced their mental wellbeing.

Our results of the duration of school or class closures showed a decreasing mental health when the number of closure days increased. The decrease per week in the duration of closure was, however, small. No studies on solely adolescents were found but a three-country study with a small sample size and parents’ answering for children and adolescents could not show a systematic pattern of psychological symptoms after two-, five- and eight-weeks quarantine [28]. Studies on adults and hospitalized patients, as summarised in a systematic review, suggest that the increased duration of quarantine is associated with negative psychological and mental health outcomes, one week or longer quarantine particularly showing the increased risk [29].

Our study was cross-sectional and does not show if mental health changed over time. All measures were reported by the pupils. Self-reports may include random errors and even bias. Concerning the duration, the number of missing answers was relatively high in both survey years and particularly in the first survey where only days, in numbers, were accepted in the answer. The reliability of the individual answers may not be optimal due to memory bias or difficulties to calculate the duration, especially if there were closures in more than one occasion. The concordance of answers on school closure (yes/no) showed a high concordance. The number of respondents was high, which diminishes random errors. The lack of anxiety measure in 2020 limits the interpretation of the effect of anxiety. Even if the estimates of our study are not exact, the direction of the effects is expected and unlikely to be biased.

In Finland, the first wave of the pandemic with school and society lockdown prevailed in March-May 2020 while our study concerned the later waves in November-December 2020 and March-May 2021. In their meta-analysis, Racine et al. [30] concluded that the prevalence of clinically elevated depression and anxiety symptoms were higher in studies collected later in the pandemic. A German study showed that adolescents’ anxiety disorder was still high on the second pandemic schoolyear compared to the pre-pandemic situation [31] and an Australian study showed that mental health symptoms were high both at the time of school closure and when the schools were reopened [16]. In our study, prevalence of daily health complaints was slightly higher in 2021 than in 2020 and if we compare these results with our first corresponding survey in May 2020 [32], a slight systematic increase can be seen. It is possible that the burden of mental health consequences among adolescents and young adults increases when the pandemic prolongs, and we cannot exclude a possibility of a further rise even after the COVID-19 pandemic has settled down to an ordinary seasonal infectious disease.

## Conclusions

Our study showed the specific negative influence of school or class closures on pupils’ mental health by comparing the groups exposed to the closure with those who were not exposed during the further waves of the pandemic. The duration of school or class closure had a small effect, too. The non-binary gender had lower mental health compared to girls and particularly to boys, but the influence of closure was not related to gender. From the point of view of pupils’ mental health, school and class closures are harmful but if these are necessary, the duration should be as short as possible. In the pandemic situation, school and class closures seem to be one factor which reduces pupils’ mental health but not the only one.

## Data Availability

The dataset analysed during the current study are not now publicly available. According to the data management plan the data are not available while reporting to the financing party is not finished, but later, the data will be available from the corresponding authors on reasonable request.
